# PHA stimulation may be useful for FDXR gene expression-based biodosimetry

**DOI:** 10.22038/ijbms.2020.42350.9997

**Published:** 2020-04

**Authors:** Habibeh Vosoughi, Hosein Azimian, Sara Khademi, Abdul-Rahim Rezaei, Maryam Najafi-Amiri, Fereshteh Vaziri-Nezamdoost, Mohammad-Taghi Bahreyni-Toossi

**Affiliations:** 1 Department of Medical Physics, Faculty of Medicine, Mashhad University of Medical Sciences, Mashhad, Iran; 2 Medical Physics Research Center, Mashhad University of Medical Sciences, Mashhad, Iran; 3 Department of Radiology Technology, School of Paramedical Sciences, Mashhad University of Medical Sciences, Mashhad, Iran; 4 Immunology Research Center, Inflammation and Inflammatory Diseases Division, Mashhad University of Medical Sciences, Mashhad, Iran

**Keywords:** Gene expression, Ionizing radiation, Low dose, Phytohemagglutinin, Radiation dose

## Abstract

**Objective(s):**

Nowadays, ionizing radiation (IR) has a significant contribution to the diagnostic and therapeutic medicine, and following that, health risks to individuals through unexpected exposure is greatly increased. Therefore, biological and molecular technology for estimation of dose (biodosimetry) is taken into consideration. In biodosimetry methods stimulation of cells to proliferation is routine to achieve more sensitivity of techniques. However, this concept has recently been challenged by new molecular methods such as gene expression analysis. This study aims to investigate the stimulation effects on gene expression biodosimetry.

**Materials and Methods:**

The blood samples were taken from15 patients who were irradiated by TC-99 MIBI, before radiopharmaceutical injection and 24 hr after injection. Lymphocytes were extracted immediately and activated by (phytohemagglutinin) PHA for 24 hr and XPA and FDXR expression levels were investigated by employing relative quantitative Real-Time PCR.

**Results:**

The results of this study show a significant increase in the FDXR expression level and a significant decrease in the XPA after stimulation of irradiated lymphocytes. Interestingly, a significant increasing trend in the FDXR expression level (at 0.05 significance level) following cell stimulation to the division was observed.

**Conclusion:**

Our results suggest that the PHA activation role in gene expression-based biodosimetry is strongly depended on the target genes and the relevant protein pathways. Finally, cell stimulation looks to be useful for some specific genes, such as FDXR, due to the increasing trend in expression and improvement of sensitivity of gene expression-based biodosimetry method.

## Introduction

With the advent of science, the ionizing radiation (IR) usage in the diagnostic and therapeutic medical sciences is increasing rapidly (1). One of the uses of ionizing radiation in medicine is nuclear medicine. According to the NCRP 93 report (1987), the diagnostic nuclear medicine contribution of total irradiation was only 4% (2), while in the NCRP 2009, this ratio has been reported 12% (3). This enhancement indicates the rapid increase of the medicine irradiation due to nuclear medicine diagnostic procedures (4). In most of nuclear medicine diagnostic procedures, including heart blood flow scan, patients receive a considerable dose compared to other imaging methods. Exposure to IR can produce well-known dose-dependent changes in biological components, including chromosomes, and proteins (5-7). According to this documentation, the ongoing effort to measure and control the irradiation dose by an appropriate dosimeter has been done (8). In any situation such as unexpected exposure to individuals where there are no physical dosimeters, alternative methods like biodosimetry play an important role. For this purpose, there is increasing concern to biological advanced techniques to estimate unknown doses in unexpected exposure to radiation. At first, chromosomal damage analysis has been established and is considered as the ‘gold standard’ of biodosimetry to estimate absorbed doses higher than 0.5 Gy of IR (9). However, most of these methods unable to detect lower doses of ionizing radiation (LDIR). It is noteworthy that diagnostic methods fall in the range of LDIR (10). Previous studies have generally been used cytogenetic tests and chromosomal abnormalities and molecular methods such as gene expression assessment are less used in this research field (11, 12). However, it should be noted that the molecular methods are more sensitive techniques than cytogenetic tests (13). When the cell is exposed to IR, it is possible that cell cycle arrest, repairing injuries or undergoing apoptosis (14, 15). For the research on IR effects many genes have been studied, but each gene uses for a specific goal and shows a different conclusion. Some studies have presented increasing evidence that various DNA repair pathways are not separated, but well interlinked. It has been suggested that non double-strand break (DSB) repair mechanisms, such as nucleotide excision repair (NER) are effectively involved in DNA repair mechanisms (16). Xeroderma pigmentosum complementation group-A (XPA) as a major factor in recognition of NER that binds to damaged DNA can be used for biodosimetry. Other gene expression biomarkers of radiation exposure have been introduced recently, Ferredoxin Reductase (FDXR) that involves in reactive oxygen species (ROS) and oxidative stresses is one of them (17-19). According to the XPA (20) and FDXR (18, 21, 22) expression level alterations, it seems these genes could be appropriate biomarkers following to LDIR. Also, it is presumed phytohemagglutinin (PHA) stimulation can be effected on the gene expression level and increased the sensitivity of gene expression-based biodosimetry methods. Finally, the present study aims to investigate XPA and FDXR expression levels following irradiation to low doses of Tc-Gamma in the PHA stimulated human peripheral blood lymphocytes and similar to Beinke’s *et al.* aims to unravel PHA activity as a prerequisite for gene expression analysis (23).

## Materials and Methods

***Selection of subjects***

In the present experimental study, at first 15 patients were selected according to the criteria for entering and leaving the study by referring to the Department of Nuclear Medicine in Ghaem Hospital of Mashhad, Iran. Patients referred to a ^99m^Tc-MIBI scan for myocardial perfusion imaging. Before Technetium-99m injection and 24 hr after injection, 3 ml of blood was taken from them (as the control group). Immediately after blood collection, lymphocytes were isolated from the Ficoll, the cells stimulated by PHA and incubated for 24 hr. The study was approved by the ethical committee of Mashhad University of Medical Sciences and all of the patients provided a consent form.

***RNA extraction and cDNA synthesize***

Blood samples (3 ml) were collected from participants using an EDTA collection tube. Peripheral blood mononuclear cells were isolated by density-gradient centrifugation using Ficoll (Cedarlane Labs, Canada). Total RNA was extracted from the whole blood using the TriPure isolation reagent (Roche Applied Science, Germany). RNA purity was quantified by spectrophotometry at 260/280 nm ratio and the integrity was confirmed by electrophoresis on a denaturing agarose gel. Then cDNA was synthesized using a commercial kit from RevertAid^TM^ First Standard cDNA Synthesis Kit (Fermentas, Germany) total mRNA, oligo-dT, dNTPs, Ribolock™ RNase inhibitor, Reaction Buffer, and RNase free water were added according to the manufacturer’s instruction.

***Real-time PCR***

Real-time PCR was performed using a master mix from SYBR® Premix Ex Taq^TM^ (Takara, Japan) and specific primers were designed and checked for FDXR, XPA and Beta-2 Microglobulin (β2M) was used as an endogenous control (Table 1)*.* Real-Time PCR protocol was run on a Real-Time PCR machine, StepOne system (Applied Biosystems), as follows: start hot temperature by 95^ ᵒ^C for 60 sec following with 40 cycles of 95 ^ᵒ^C (10 sec) and 60 ^ᵒ^C (30 sec). Raw data were calculated using a relative quantitative standard curve method. 

***Statistical analysis***

After evaluation of the normality distribution of the data, One Way ANOVA under SPSS software version 18, and GraphPad Prism, version 7.01was used to analyze the data. After evaluation of the normality distribution of the data, the statistical significance of differences between groups was analyzed by Student’s paired t-test under SPSS software version 18 and GraphPad Prism, version 7.01. A value of *P*<0.05 was considered statistically significant difference.

## Results

The received doses following technetium-99m methoxyisobutylisonitrile (^99m^Tc-MIBI) injection for each patient are shown in Table 2.

The gene expression assessments were performed for each patient separately, and the consequences have been shown in Figure 2. The results of the present investigation are evident that a significant up-regulation of the FDXR gene has been induced in the irradiated group. While expression of the XPA gene was found to be down-regulated after irradiation by ^99m^Tc.

**Figure 1:**
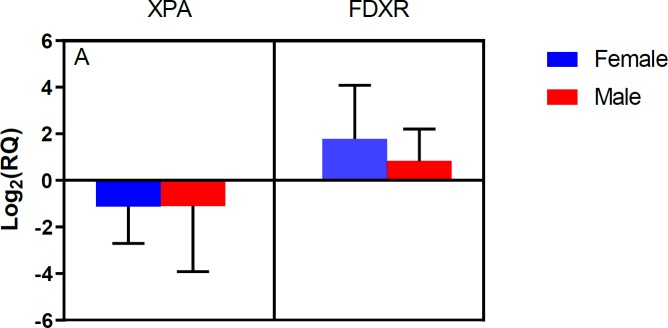
Relative expression of XPA and FDXR genes following irradiation considering patients’ gender

FDXR: Ferredoxin Reductase; XPA: Xeroderma pigmentosum complementation group-A

**Table1:** Primers used for genes in SYBR green real-time PCR

Gene	Primer sequence (5` to 3`)	Length	Tm
β2M	Forward: GTATGCCTGCCGTGTGAAC	19	59
	Reverse: AACCTCCATGATGCTGCTTAC	21	59
FDXR	Forward: CATAGCCACAACCATGACTGACAG	24	58
	Reverse: CCACCTCCTCGGCATCCA	18	58.8
XPA	Forward: CTGGAGGCATGGCTAATG	18	56
	Reverse: CAAATTCCATAACAGGTC	18	57

β2M: Beta-2 Microglobulin; FDXR: Ferredoxin Reductase; XPA: Xeroderma pigmentosum complementation group-A

**Table 2:** The received doses following 99mTc-MIBI injection for each patient

		Gender	Age	A_0/m_	Effective dose (mSv)
1		Female	62	0.227	4.646
2		Male	53	0.277	5.699
3		Male	65	0.277	5.669
4		Female	50	0.298	6.099
5		Male	51	0.295	6.038
6		Female	56	0.350	7.164
7		Female	61	0.345	7.061
8		Male	65	0.365	7.471
9		Male	56	0.250	5.117
10		Male	57	0.285	5.833
11		Male	55	0.294	6.017
12		Female	50	0.235	4.810
13		Male	64	0.280	5.731
14		Female	60	0.303	6.202
15		Female	45	0.258	5.281

Since the results of 2 samples were flipping data (samples No 10. and 15.), which may have an abnormal radiation-sensitivity, have been deleted from data analysis. Figure 1 illustrates the effects of gender on the gene expression levels. According to the statistical analysis, there was no significant difference between the female and male groups in the expression levels of the selected genes (*P*-value for XPA=0.51 and FXDR=0.12).

**Figure 2:**
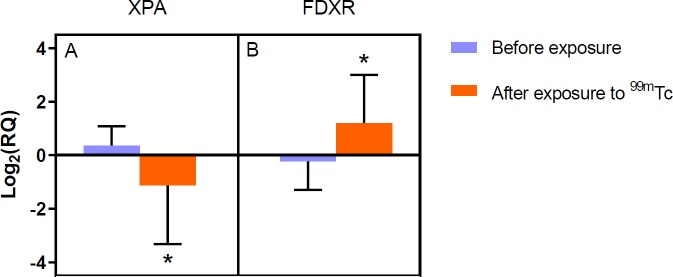
Alterations in the levels of XPA (A) and FDXR (B) gene expression following irradiation to low doses of ^99m^Tc in the PHA stimulated human peripheral blood lymphocytes. The error bars show standard deviations for each group. Significance of induced changes in irradiated groups in comparison with the control group is implied by * (*P*-value<0.05)

FDXR: Ferredoxin reductase; XPA: Xeroderma pigmentosum complementation group-A

**Figure 3:**
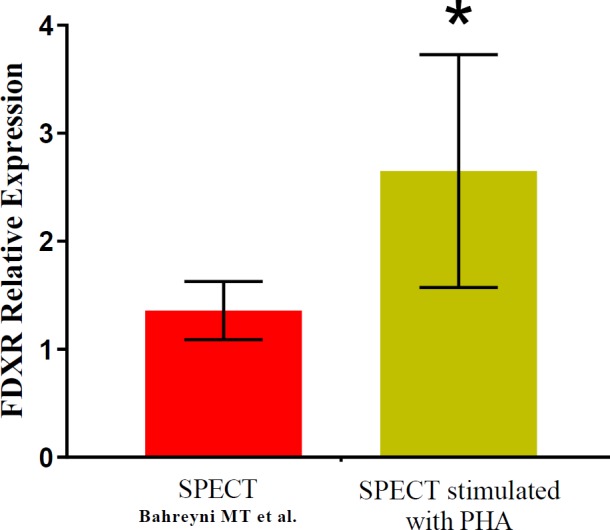
Relative expression of FDXR in blood samples were taken at 24 hr after radiation exposure from patients exposed to SPECT (with permission. Copyright © 2018, mums.ac.ir) (35) using Web Plot Digitizer in comparison with PHA stimulated samples from SPECT patients. Each bar represents mean value for the patients and Error bars show standard deviation. *represent significant differences with other group

FDXR: Ferredoxin Reductase; SPECT: Single Photon Emission Computed Tomography

## Discussion

A large number of studies have been investigated high doses and the detrimental effects in this range have been known well (24-26). But there is little contradictory scientific evidence at low doses, such as adaptive response (27-29), radiation hormesis (30) and cancer induction (31). These responses depend on several factors, including cell type, specific genes mutation and cell cycle phase (32). As previously mentioned, some studies have revealed increasing evidence that various repair pathways are effectively involved in DNA repair mechanisms. In other words, DSB can be formed as the consequence of the production of SSB or nucleotide excision opposite each other on the two strands of DNA. One of these studies found that suppressed expression of DNA repair genes (except DSB), influenced the yield of ionizing radiation-induced cytogenetic aberrations, suggesting that this gene is highly involved in DSB repair (33). On the other hand, the total yield of nucleotide excision in mammalian cells should be on the order of 2000/cell/Gy and DSBs taken as 40/cell/Gy, therefore it seems NER to be, at least partly, an acceptable biomarker of DNA damages (34). Thus, the up-regulation of XPA gene expression even following low doses of Gamma radiation is an indication of DNA repair activation (35). Also, XPA overexpression in non-stimulated peripheral blood lymphocytes (PBLs) has been reported in several studies (20). Mayer *et al.* observed stimulation of PBLs to proliferate did not affect their capacity to repair radiation-induced DNA damage but in our study XPA as a major factor in NER recognition, suppressed significantly. They revealed no difference, neither in the rate of radiation-induced DNA damage nor in DNA repair capacity between PHA-stimulated and non-stimulated PBLs (36). In contrast to earlier findings, the results of this article show that the XPA gene expression level of PHA stimulated PBLs down-regulated after irradiation to LDIR. Zhang *et al.* revealed down-regulation of the XPA gene expression level, in agreement with our findings, resulting in significantly enhanced cell cycle progression (33). These data suggest that DNA repair proteins needed for the repair of IR-induced damage, are already present in G0 cells at sufficient amounts and do not need to be stimulated to start cycling. PHA stimulation is widely used in biodosimetry assays to shift a certain fraction of lymphocytes from G0 into the G1 phase of the cell cycle. These methods are limited by the time-consuming PHA mediated lymphocyte activation (48 hr) and saturation of the dose-response curve in 4 Gy where the curve reaches a plateau. Some studies have been tried to develop a rapid estimation of absorbed dose within 2 hr when compared with the analysis at metaphase, which presupposes a 2-day delay for lymphocyte culture (37, 38). Gene expression-based biodosimetry is considered one of these methods, which can provide accurate and rapid dosimetry. Dose estimates based on FDXR, using qRT-PCR is introduced as a precise method for biodosimetry in several studies (19, 39, 40). PHA-treatment would result in increased cell metabolism with stimulating cell proliferation and following that activate many pathways in cell cycle progression. It is presumed that cell stimulation can be influenced on the genes expression and the sensitivity of gene expression-based biodosimetry methods may be enhanced. As shown in Figure 2B, expression of FDXR increased 24 hr following to *in-vivo* exposure and after PHA stimulation. To compare, the FDXR expression levels in non-stimulated PBLs have been reproduced (with permission) in Figure 3. 

FDXR expression extracted from SPECT patients described by Bahreyni-Toossi *et al.* (35) (with permission. Copyright © 2018, mums.ac.ir) and CT-scan and fluoroscopy previously reported by O’Brien* et al. *(19) (with permission. Copyright © 2018, Springer Nature) using Web Plot Digitizer v. 4.2 (41) and finally is analyzed by GraphPad Prism, version 7.01. The FDXR level has been significantly increased after using PHA in comparison with non-stimulated PBLs 24 hr following to exposure from patients exposed to medical imaging procedures including SPECT (shown in Figure 3), CT-scan, and fluoroscopy (data not shown).

Stimulation, in order to cell division as previously reported in the many cases(42-44), looks to be useful for gene expression-based biodosimetry, however, this depends on the protein function that is coded by a specific gene which is chosen as a biomarker. On the other hand, some cellular pathways in molecular biodosimetry may benefit from stimulation.

## Conclusion

The purpose of this study was to investigate the PHA activation role in gene expression-based biodosimetry in the LDIR region. First, our results do not support a general increase in DNA repair activity of PBLs by PHA stimulation. Secondly, the use of stimulated PBLs in molecular studies on IR-induced DNA damage seems not to be mandatory, when the DNA repair mechanism is the endpoint. Thirdly, cell stimulation in order to induce division looks to be useful for gene expression-based biodosimetry with some specific genes, such as FDXR. Taken together, our results suggest that PHA stimulation benefits in gene expression-based biodosimetry are strongly dependent on the target genes and the relevant protein pathways. 
